# Supplementary figures and images for: Intravenous Administration of Mesenchymal Stem Cell-Derived Exosome Alleviates Spinal Cord Injury by Regulating Neutrophil Extracellular Trap Formation through Exosomal miR-125a-3p
Source: Int J Mol Sci. 2024 Feb 18;25(4):2406. doi: 10.3390/ijms25042406 (PMC10889446; doi:10.3390/ijms25042406)

## Slide 1
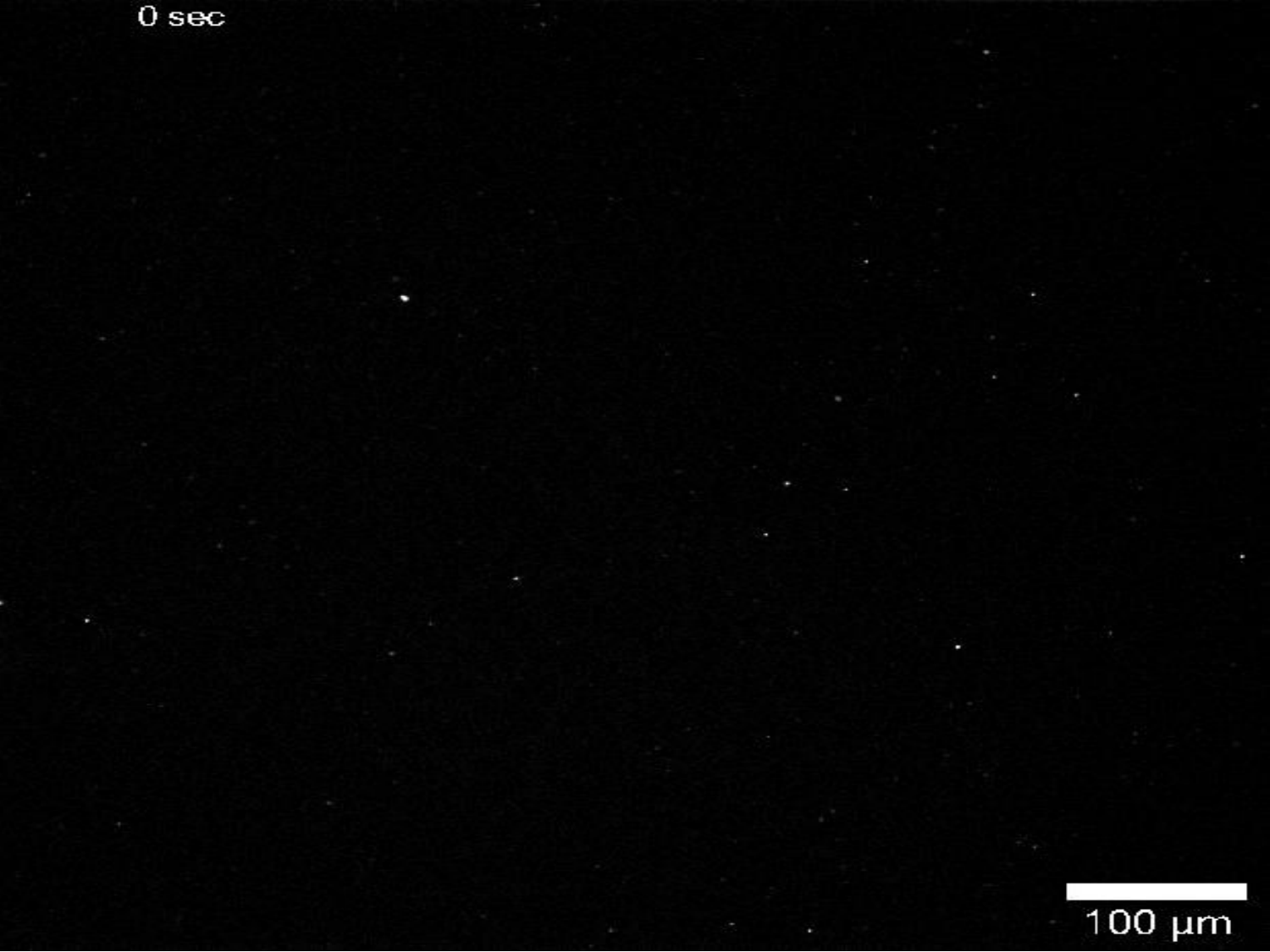

Supplement: Supplementary file 1 [file ijms-25-02406-s001.zip › Supplementary Video.pptx]
